# Tribute to Dr Andre Marais: 1976–2020

**DOI:** 10.4102/safp.v62i1.5185

**Published:** 2020-07-22

**Authors:** Indiran Govender

**Affiliations:** 1 Department of Family Medicine, University of Pretoria and Kalafong Hospital, Pretoria, South Africa

It is with profound grief that I write this obituary for my dear friend Dr Andre Marais. Dr Andre Marais passed away on 12 June 2020. He completed matric at Afrikaanse Hoërskool Germiston. After qualifying, he worked as an intern and community service doctor at Dr George Mukhari Academic Hospital. He also worked at Tamarisk Pharmacy, Biomox Pharmaceuticals, Arrie Nel Pharmacy Group and Medunsa campus of the University of Limpopo, had his own specialist practice and lectured at the University of Pretoria.

Since 1 September 2013, Dr Marais was a lecturer at the University of Pretoria’s Department of Pharmacology. He held the BPharm, MBChB and MSc (Pharmacology) degrees and was a registered clinical pharmacologist.

He was dedicated to clinical services and academic teaching and learning. He served as vice president of the South African Society for Basic and Clinical Pharmacology. He was the deputy chair of the South African Medical Association’s Social and Ethics Committee, a council member of the South African Academy of Family Physicians and National Councillor of the College of Clinical Pharmacologists (Colleges of Medicine of South Africa). At the University of Pretoria, he developed and coordinated the Regulatory Affairs in the BSc Honours Pharmacology programme, which was the first and only one of its kind in the country.

Andre will be remembered as a kind, humble, soft-spoken person, who was always willing to help. He will always be remembered for his generosity. The medical fraternity has lost a dear friend, colleague and wonderful clinician who gave off his time to serve our society. Our deepest condolences to all his friends, colleagues, wife and children.

May his soul rest in peace.

